# Modern contraception utilization and associated factors among all women aged 15–49 in Ethiopia: evidence from the 2019 Ethiopian Mini Demographic and Health Survey

**DOI:** 10.1186/s12905-023-02203-8

**Published:** 2023-02-09

**Authors:** Girum Taye Zeleke, Theodros Getachew Zemedu

**Affiliations:** Health System and Reproductive Health Research Directorate, Ethiopian Public Health Institute, Addis Ababa, Ethiopia

**Keywords:** Ethiopia, Mini-DHS, Modern contraceptives

## Abstract

**Background:**

The use of contraceptive is key in reducing unsafe abortion from unintended pregnancies, infant mortality, adolescent pregnancies, slowing population growth and helps to prevent HIV/AIDS. However, less than one-third of women within reproductive age in Ethiopia uses modern contraceptive methods. Hence, this study aimed to determine the prevalence of modern contraceptive utilization and to identify potential factors on use of modern contraceptive method.

**Methods:**

Data from 2019 Ethiopian Mini Demographic and Health Survey were used in this analysis. A total of 8885 women within the ages of 15–49 years across 305 enumeration areas in nine regions and two city administrations were included in the analysis.

Multivariable logistic regression model were applied to examine the association between women’s background characteristics and modern contraceptives utilization.

**Results:**

Only 28.1% of all women used modern contraceptives. About 40% of the modern contraceptive users were between age of 25–29 and 30–34 years. There was significant association between women’s age, level of education, region, religion, parity, wealth quintile and marital status on use of modern contraceptives. Women who were married and living with partners were about 20 (AOR = 19.91, 95% CI: 14.27, 27.78) and 24 (AOR = 23.51, 95% CI: 14.66, 37.72) times more likely to use modern contraceptives compared to sexually active unmarried women.

**Conclusion:**

The study showed that the use of modern contraceptive method is not adequate and it is also influenced by socio-demographic and economic characteristics of women in Ethiopia. Therefore, increasing the awareness of women to use modern contraceptive methods is vital. We suggest that there is a need to improve the service in women age above 39 years, women in Afar, Somali, Harari and Diredawa regions; and protestant, Muslim and traditional religion followers.

## Background

Family planning refers to a decision of couple that is intended to limit or space the number of children they will have through the utilization of contraceptive methods. Modern contraceptive methods include the pill, IUD, injectable, implants, male condom, emergency contraception, standard day’s method (SDM), and lactational amenorrhea method (LAM) and other modern method [1].

The use of contraceptive is key strategy in reducing unsafe abortion and unintended pregnancies [2]. Strengthening family planning services is crucial to improving maternal death. Studies have showed that up to 44% of maternal deaths could have been averted through the use of family planning services [3]. Evidence has also indicated that short birth spacing puts, which might be prevented by the use of family planning, the mother and the preceding child at high risk of morbidity and mortality [4]. In response to this, increasing access to family planning services has become a globally recognized public health intervention.

The world’s population by 2015 was estimated at 7.3 billion and projected to be 8.5 billion in 2030 [5]. This world population projection increment mainly depends on high total fertility rate of countries including Ethiopia in which it had a total fertility rate of 4.6 children per woman in 2016 [6]. The population of Ethiopia was estimated in 2007 at about 73 million and it is projected to be about 116 million by 2030 [7].

Globally, from the total of 1.9 billion women in reproductive age, 1.1 billion have a need to use family planning Of these women, 842 million are users of modern methods of contraception [8].

Sub-Saharan Africa has the highest fertility rate in the world, with the highest unmet need for family planning. Yet, there is a lack of knowledge about the determinants for non-utilization of modern contraceptive methods among women of reproductive age [4].

The recent Ethiopian intrim Demographic and Health Survey have shown inadequate uptake of contraceptive methods and high-unmet needs compared with the target set on the Health Sector Transformation Plan (HSTP)-I among women aged above 39 years. The survey reported about 29% of all women between age 15–49 years used any (either modern or traditional) contraception method and only 28% of the women utilized modern contraceptive methods [9].

Multiple factors could contribute for the utilization of family planning services. In previous studies in Ethiopia (socio-economic status, Urban/Rural location and regions) [10], Nigeria (age, parity and partner’s disapproval) [11, 12], Malawi (wealth quintal, educational level and fertility intention) [13] and Ghana (cultural belief, and luck of awareness, fear of side effects, and misconceptions) [14] have shown influence on uptake of modern contraceptive methods.

Given the above conflicting evidenced on factors contributing for family planning utilization, this study aimed to determine the prevalence of modern contraceptive utilization and the potential factors influencing the use of modern contraceptive method among Ethiopian women aged 15–49 years from the national data source.

## Methods

### Study design and setting

Secondary data from the 2019 Ethiopian mini Demographic and Health Survey were used in this analysis. It is a national representative data that was collected through a community-based cross-sectional survey from March 21, 2019 to June 28, 2019.

*Sampling procedure and study population* The sample for the survey was stratified and selected in two stages.

In the first stage, 305 enumeration areas (EAs) from a frame of all EAs created for the 2019 Ethiopia Population and Housing Census by Central Statistical Agency selected with probability proportional to EAs size.

In the second stage, a fixed number of 30 households per EAs selected with an equal probability systematic selection from the newly created household listing. All women age 15–49 who were either permanent residents of the selected households or visitors who slept in the household the night before the survey were eligible for interview. Detailed method has been previously reported [9]. The study population for this analysis was all reproductive age women in Ethiopia.

### Study variables

The dependent variable is the status of using of modern contraceptive methods during the survey, which categorized as ‘Yes’ for those who uses either female sterilization, male sterilization, PILL, IUD, injectable, implants, male condom, female condom, emergency contraception, standard days method (SDM), lactational amenorrhoea (LAM) and other modern method, while “No” for those who used either rhythm, withdrawal, other traditional method users, and none users of family planning methods.

And respondent age, residence, region, education and wealth quintile included as independent variable.

### Data analysis

After permission secured from MEASURE DHS, the data for this analysis was accessed and downloaded in SPSS format from the website. To summarize initially the study variables alone and to explore association between study variables with utilization of modern contraceptive methods; frequency count and proportion were implemented. For these analyses, survey-weighting variable applied to produce appropriate representation of family planning information and associated factors.. To examine the unadjusted association between use of modern contraceptive methods and study variables, univariable logistic regression model applied. To examine the overall final adjusted association, variables indicated association at 2% level of significance in bivariable logistic regression analysis were included in a multivariable logistic regression. To check multicollinearity between independent variables and goodness of the model fit, Spearman rank correlation and the Log likelihood test were implemented, respectively. Finally, measure of associations of the study variables on use of modern contraceptive methods examined using adjusted Odds Ratio (OR) at 95% confidence interval (CI). All the analyses done using SPSS Version 20.

## Results

### Women socio-demographic and economic characteristics

From the total 8885 women in the survey, those in age group 15–19 years were the highest proportion (24.9%) followed by women in age group 25–29 (18.8%). Most of women in the survey were completed primary level of education (41.7%) while 5.7% of the women have more than secondary educational level.

Generally, compared to their respective categories more proportion of women in this survey were from rural areas (67.8%) and Oromia regional state (37.7%); Orthodox religion (41.5%); married (64.6%); with zero parity (32.8%) and in the highest wealth quintile (25.7%) (Table 1).

**Table 1:** Socio-demographic and economic characteristics of women (aged 15–49 years) in Ethiopia, min DHS 2019

Variables	Frequency	Weighted percentage
*Age group*		
15–19	2100	24.9
20–24	1578	16.7
25–29	1752	18.8
30–34	1166	13.1
35–39	1037	12.0
40–44	714	8.3
45–49	538	6.3
*Educational level*		
No education	3640	40.4
Primary	3345	41.7
Secondary	1149	12.2
More than Secondary	751	5.7
*Residence*		
Urban	2951	32.2
Rural	5934	67.8
*Region*		
Tigray	733	7.1
Afar	641	1.0
Amhara	948	22.8
Oromia	1052	37.7
Somali	640	4.7
Benishangul-Gumuz	747	1.1
SNNPR	1008	19.2
Gambela	723	0.5
Harari	763	0.3
Addis Ababa	818	5.0
Dire Dawa	812	0.7
*Religion*		
Orthodox	3374	41.5
Catholic	78	0.5
Protestant	1711	27.4
Muslim	3635	29.5
Traditional	60	0.9
Other	27	0.2
*Marital status*		
Single	2300	26.2
Married	5613	64.6
Living with partner	129	1.4
Widowed	227	2.1
Divorced	424	4.2
Separated	192	1.5
*Parity*		
None	2897	32.8
1 to 2	2404	25.2
3 to 4	1705	19.1
5 and above	1879	22.9
*Wealth quintile*		
Lowest	2031	16.2
Second	1341	18.2
Middle	1268	18.8
Fourth	1344	21.1
Highest	2901	25.7
Total	8885	100

#### Prevalence of modern contraceptive utilization among women

Findings in 2019 Ethiopian Mini Demographic and Health survey shows, modern contraceptives were used by 28.1% of women, of which 18.7% were injectable, 6% implants, 1.4% pill, 1% IUD, 0.4% LAM, 0.3% female sterilization and 0.2% male condom and 0.1% SDM.

In this modern contraceptive utilization, disparity has been observed by sociodemographic and socio-economic characteristics of women. Only 2.1% of single and 2.2% of widowed women utilized modern contraceptive method, whereas, 12.7% separated 14.6% divorced, 40.4% married and 43% living with their partners women utilized modern contraceptive methods, respectively.

About 41% of women aged 25–29 years utilized modern contraceptive followed by women in the age group 30–34 (39.5%), 20–24 (34.7%) and 35–39 (34.1%) (Table 2).

**Table 2:** Percent distribution of contraceptive utilization type by socio-demographic and economic characteristics of women (aged 15–49 years) in Ethiopia, min DHS 2019

Variables	Use of modern FP n (weighted %)	Traditional or non FP users n (weighted %)
*Age group*		
15–19	164 (9.4)	1936 (90.6)
20–24	435 (34.7)	1143 (65.3)
25–29	568 (40.5)	1184 (59.5)
30–34	380 (39.5)	786 (60.5)
35–39	305 (34.1)	732 (65.9)
40–44	150 (26.9)	564 (73.1)
45–49	59 (13.7)	479 (86.3)
*Educational level*		
No education	759 (27.6)	2881 (72.4)
Primary	841 (28.6)	2504 (71.4)
Secondary	267 (26.3)	882 (73.7)
More than Secondary	194 (31.0)	557 (69.0)
*Residence*		
Urban	697 (29.4)	2254 (70.6)
Rural	1364 (27.4)	4570 (72.6)
*Region*		
Tigray	183 (26.2)	550 (73.8)
Afar	63 (11.6)	578 (88.4)
Amhara	319 (34.4)	629 (65.6)
Oromia	288 (27.5)	764 (72.5)
Somali	15 (2.9)	625 (97.1)
Benishangul-Gumuz	202 (26.3)	545 (73.7)
SNNPR	313 (31.1)	695 (68.9)
Gambela	176 (22.5)	547 (77.5)
Harari	152 (18.5)	611 (81.5)
Addis Ababa	205 (24.7)	613 (75.3)
Dire Dawa	145 (18.5)	667 (81.5)
*Religion*		
Orthodox	998 (31.7)	2376 (68.3)
Catholic	20 (44.7)	58 (55.3)
Protestant	465 (31.3)	1246 (68.7)
Muslim	562 (20.1)	3073 (79.9)
Traditional	9 (20.5)	51 (79.5)
Other	7 (25.7)	20 (73.3)
*Marital status*		
Single	2245 (2.1)	55 (97.9)
Married	3737 (40.4)	1876 (59.6)
Living with partner	78 (43.0)	51 (57.0)
Widowed	221 (2.2)	6 (97.8)
Divorced	376 (14.6)	48 (85.4)
Separated	167 (12.7)	25 (87.3)
*Parity*		
None	225 (8.3)	2672 (91.7)
1 to 2	891 (45.5)	1513 (54.5)
3 to 4	534 (38.4)	1171 (61.6)
5 and above	411 (28.7)	1468 (71.3)
*Wealth quintile*		
Lowest	231 (20.2)	1800 (79.8)
Second	326 (25.7)	1015 (74.3)
Middle	368 (31.7)	900 (68.3)
Fourth	384 (28.8)	960 (71.2)
Highest	752 (31.5)	2149 (68.5)
Total	2061 (28.1%)	6824 (71.9%)

#### Factors associated with the utilization of modern contraceptive method in women

The univariable logistic regression analysis showed a significant association (p < 0.001) between modern contraceptive utilization with women’s age group, educational level, region, religion, parity, wealth quintile and marital status. These variables were subsequently included in the multivariable logistic regression model.

The multivariable logistic regression showed that, women in age group 20–24 (aOR:1.66, 95% CI:1.31–2.11), 25–29 (aOR:1.54, 95% CI: 1.21–1.97), 30–34 (aOR:1.61, 95% CI: 1.22–2.11), 35–39 (aOR:1.34, 95% CI: 1.01–1.78) were more likely utilized modern contraceptive method while women in age group 45–49 (aOR:0.40, 95% CI:0.27–0.59) were less likely used modern contraceptive method compared to those in age group 15–19 during the survey. Comparison by level of education showed, women with primary levels of education (aOR: 1.47, 95% CI: 1.27–1.7) and with secondary level of education (aOR: 1.29, 95% CI: 1.04–1.59) were more likely utilized modern contraceptive compared to those who had no education.

Regarding modern contraceptive use in different regions, women in Afar (aOR:0.60, 95% C.I:0.41–0.86) and Somali (aOR:0.16, 95% CI: 0.09–0.29) were less likely utilized modern contraceptive compared to those women in Tigray region, while those women in Amhara (aOR:1.82, 95% CI: 1.42–2.33), Oromia (aOR:1.62, 95% C.I: 1.24–2.12), Benishangul-Gumuz (aOR:1.57, 95% C.I: 1.19–2.06) and SNNPR (aOR:1.84, 95% CI: 1.39–2.43) were more likely used modern contraceptive compared to those women in Tigray region.

Regarding wealth quintile, women in the highest wealth quintile (aOR: 2.91, 95% CI: 2.33–3.63), fourth (aOR: 2.15, 95% C.I: 1.74–2.65), middle (aOR: 1.94, 95% CI: 1.57–2.39), second (aOR: 1.65, 95% CI: 1.34–2.04) were more likely utilized modern contraceptive compared to those in the lowest wealth quintile (Table 3).

**Table 3:** Crude and adjusted Odds Ratio for modern contraceptive use among women (aged 15–49 years) in Ethiopia, min DHS 2019

Variables	Crude estimates		Adjusted estimates	
	Odds ratio	95% CI	Odds ratio	95% CI
*Age group*				
15–19	1		1	
20–24	4.49	3.70–5.45	1.66	1.31–2.11*
25–29	5.66	4.69–6.84	1.54	1.21–1.97*
30–34	5.71	4.67–6.98	1.61	1.22–2.11*
35–39	4.92	3.99–6.06	1.34	1.01–1.78*
40–44	3.14	2.47–3.99	0.92	0.67–1.26
45–49	1.45	1.06–1.99	0.40	0.27–0.59*
*Educational level*				
No education	1		1	
Primary	1.28	1.14–1.43	1.47	1.27–1.70*
Secondary	1.15	0.98–1.35	1.29	1.04–1.59*
More than Secondary	1.32	1.10–1.59	1.28	0.99–1.64
*Residence*				
Urban	1			
Rural	0.97**	0.87–1.07	–	–
*Region*				
Tigray	1		1	
Afar	0.33	0.24–0.45	0.60	0.41–0.86*
Amhara	1.52	1.23–1.89	1.82	1.42–2.33*
Oromia	1.13	0.91–1.41	1.62	1.24–2.12*
Somali	0.07	0.04–0.12	0.16	0.09–0.29*
Benishangul-Gumuz	1.11	0.88–1.41	1.57	1.19–2.06*
SNNPR	1.35	1.09–1.68	1.84	1.39–2.43*
Gambela	0.97	0.76–1.23	1.13	0.84–1.51
Harari	0.75	0.59–0.95	0.77	0.57–1.04
Addis Ababa	1.01	0.80–1.27	1.01	0.75–1.35
Dire Dawa	0.65	0.51–0.84	0.80	0.59–1.09
*Religion*				
Orthodox	1		1	
Catholic	0.82	0.49–1.37	1.16	0.62–2.15
Protestant	0.89	0.78–1.01	0.71	0.59–0.85*
Muslim	0.44	0.39–0.49	0.54	0.46–0.64*
Traditional	0.42	0.21–0.86	0.40	0.19–0.86*
Other	0.83	0.35–1.98	0.85	0.32–2.28
*Parity*				
None	1		1	
1 to 2	6.99	5.96–8.20	1.64	1.31–2.04*
3 to 4	5.42	4.57–6.42	1.44	1.12–1.86*
5 and above	3.33	2.79–3.96	1.25	0.95–1.66
*Wealth quintile*				
Lowest	1		1	
Second	2.50	2.08–3.01	1.65	1.34–2.04*
Middle	3.19	2.65–3.83	1.94	1.57–2.39*
Fourth	3.12	2.60–3.74	2.15	1.74–2.65*
Highest	2.73	2.32–3.20	2.91	2.33–3.63*
*Marital status*				
Single	1		1	
Married	20.49	15.59–26.93	19.91	14.27–27.78*
Living with partner	26.69	17.14–41.56	23.51	14.66–37.72*
Widowed	1.11	0.47–2.60	1.46	0.60–3.55
Divorced	5.21	3.49–7.79	4.08	2.61–6.37*
Separated	6.11	3.71–10.06	4.23	2.47–7.23*

*Statistically significant at 5% level

**Statistically not significant at 20% level (p-value = 0.51)

## Discussion

Twenty eight percent of women in reproductive age who were included in the study used modern contraceptive method. This study also identified women’s age, educational level, region, religion, parity, wealth quintal and marital status to be significant factors that were influencing modern contraceptive use.

This study used national and regional representative data to reflect the prevalence of the reproductive age women modern contraceptive utilization and its possible associated factors. The data were collected through adapted DHS program’s standard questionnaires.

Findings in 2019 Ethiopian Mini Demographic and Health survey shows that uptake of modern contraceptive method at national level has improved to 28.1% from previous years Ethiopian Demographic and Health Survey, that is 9.7% in 2005, 18.7% in 2011 and 24.9% in 2016 [6, 9, 15, 16]. This could be due to the reason that, in the recent survey, number of women received counseling from a health professional about modern contraceptives health benefits increased slowly through time.

Although improvements have been shown, the contraceptive acceptance rate in Ethiopia is still beyond its HSTP target for the same year [17]. In order to achieve this target in the remaining 2 years, interventions like health education on advantage of contraceptive use, community mobilization and improvement in family planning accessibility are among the key strategies. The likelihood of modern contraceptive use was higher among married and living with partner women compared to those who are unmarried but sexually active. This could be from partner support on contraceptive use [18] or women’s independency in making beneficial reproductive health decisions [19].

Our study findings indicated that women from highest wealth quintal, primary level of education, SNNPR/Amhara regions and 1 to 2 parity being more likely to use modern contraceptive compared to women in lowest wealth quintal, with no education, from Tigray and with no parity, respectively. Our finding is supported with a multi-country study conducted by health policy initiative in 2007 across 47 developing countries revealed the inequalities in the use of family planning observed among different women groups [20]. This is due to the fact that women from rural areas might not easily access infrastructure to access the facility and availability of modern contraceptive service. In addition, it may be the misperception and myths about modern contraceptives especially by women with no education. This study revealed that women whose age was between 20 and 24 was more likely to utilize modern contraceptive methods as compared to women whose age were between 15 and 19. Similarly, results from a study in Angola also showed that a woman whose age was from 15 to 19 years less likely practiced modern contraceptive utilization which is consistent with our study [21]. This may be due to they are economically dependent and do not want to have a child and use modern contraceptive to delay pregnancy.

This study has the following strength. The study uses nationally representative datasets collected using standardized methodologies and instruments. However, the following limitations should be considered. The analysis was limited to include available variables in the dataset and did not include some aspects. This limitation arose because the study used secondary data.

The findings of our study implies to improve the gap of up taking modern contraceptive, the policy makers who are working in the area need to give more attention for reproductive age women from lowest quintile, for Afar and Somali regions and with no education.

This study has a number of implication as it indicated inadequate family planning utilization. The government waived this service in the country. However, a national survey indicated that private facilities were less likely to provide the service than their public counterpart [22].

Therefore, ministry should support and reinforce the service availability at private facilities as well. In addition, the supply system for family planning should be strengthened to avoid stock out in the facilities which will help to narrow the unmet need.

And we recommend the policy makers and non-governmental organizations working on Family Planning to facilitate and support the provision of health education to change traditional and religious attitudes towards considering more children as benefit of the family at district health offices.

## Conclusion

Modern contraceptive utilization in Ethiopian women in the reproductive age is found to be low. This study also pointed out a disparity in modern contraceptive use among regions, and wealth quintiles. In addition, we found low utilization of modern contraceptive among unmarried women, 15–19 age group, and Muslim women. Women’s age, educational level, region, religion, parity, wealth quintal and marital status associated with modern contraceptive use. Therefore, increasing the awareness of women to use modern contraceptive methods is vital, which can be achieved by public health intervention that focus on women in age greater than 39 years, women in Afar, Somali, Harari and Diredawa regions; and protestant, muslim and traditional religion followers.

## Data Availability

Data are available in a public, open access repository. The data for this study were sourced from Demographic and Health Survey and are available at http://www.dhsprogram.com/data/available-datasets.cfm. With file name “ETIR81SV.ZIP” in the link https://dhsprogram.com/data/dataset/Ethiopia_Interim-DHS_2019.cfm?flag=0.

## Abbreviations

aOR: Adjusted Odds Ratio
CI: Confidence interval
CSA: Central Statistical Agency
DHS: Demographic and Health Survey
EPHI: Ethiopian Public Health Institute
FP: Family planning
HSTP: Health Sector Transformation Plan
IRB: Institutional Review Board
LAM: Lactational amenorrhoea
MoH: Ministry of Health
SDM: Standard days method
SPSS: Statistical package for social science
SNNPR: Southern Nations and Nationalities Peoples Region
